# Genetic Variation between Asian and Mediterranean Populations of Cucurbit Aphid-Borne Yellows Virus

**DOI:** 10.3390/v15081714

**Published:** 2023-08-09

**Authors:** Parastoo Pouraziz, Milad Yousefi, Adyatma Irawan Santosa, Davoud Koolivand

**Affiliations:** 1 Department of Plant Protection, Faculty of Agriculture, University of Zanjan, Zanjan 45371-38111, Irankoolivand@znu.ac.ir (D.K.); 2 Department of Plant Protection, Faculty of Agriculture, Universitas Gadjah Mada, Yogyakarta 55281, Indonesia

**Keywords:** gene flow, molecular characterization, phylogroup, population genetics, recombination

## Abstract

Viral symptoms, such as yellowing, leaf deformation, mottling, vein clearing, and reduced yield, were observed in cucurbits in Iran. This study aimed to detect the main suspected causal agent, cucurbit aphid-borne yellows virus (CABYV), in Iran and analyze the genetic diversity among isolates. Two hundred samples were collected from different growing areas between 2019 and 2022. PCR amplification was performed on the P3 and P4 genes. The sequences of 18 Iranian isolates were obtained and deposited in GenBank. Recombination, phylogenetic, and population genetics studies were then carried out for the complete genome and all ORFs sequences, together with other isolates in GenBank. The nucleotide identities of the overlapped ORF3/4 sequences of Iranian isolates were 94.8 to 99.5% among themselves, and with other tested isolates ranging from 94.3 to 99.3%. Phylogenetic trees based on the complete genome and the overlapped ORF3/4 showed two major clades, namely Asian and Mediterranean, and the new isolates from Iran were positioned in both clades. The obtained results also suggest that all the genes and two clades of CABYV populations were under negative selection pressure. Furthermore, rare gene flow between these two clades (*F*_ST_ > 0.33) confirmed the high genetic separation among them.

## 1. Introduction

Cucurbitaceae is a prominent family of vegetables that is extensively cultivated in specific regions worldwide. It comprises several economically significant species, including watermelon (*Citrullus lanatus*), cucumber (*Cucumis sativus*), melon (*Cucumis melo*), and zucchini (*Cucurbita pepo*). These cucurbit species are predominantly grown during summer season, particularly in the Mediterranean. Plant viruses pose a severe threat to the cucurbit crops, causing over US$ 30 billion worth of damage annually [1]. In Iran, for instance, approximately 35 viruses have been reported to infect cucurbit plants, including zucchini yellow mosaic virus (ZYMV), cucumber mosaic virus (CMV), watermelon mosaic virus (WMV), and cucurbit aphid-borne yellows virus (CABYV) [2,3,4].

CABYV, in particular, is one of the most prevalent viruses affecting cucurbit crops, especially in Mediterranean and subtropical climates [5]. The virus is a member of the *Polerovirus* genus, which was recently transferred from the outdated *Luteoviridae* to *Solemoviridae* family [6]. It was first reported in France in 1995 and has since detected worldwide, inducing a significant reduction in cucurbit production [7]. Under field conditions, the main symptoms caused by CABYV include the yellowing and thickening of older leaves. The symptoms vary depending on the season and plant cultivars [8]. Additionally, the virus causes flowers to die during flowering, resulting in a reduction in the number of fruits, although the quality of the fruit in general is not affected [9]. CABYV is mainly found in phloem cells and is transmitted in a circulatory, persistent manner by *Aphis gossypii* and *Myzus persicae* [5,10].

The viral genome is a positive single-stranded RNA approximately 5.7 kb in size, containing six open reading frames (ORFs). ORFs P0 and P1 encode a suppressor of post-transcriptional gene silencing (PTGS) and serine proteases with genome-linked viral proteins (VPgs), respectively, both of which are translated from the RNA genome. ORFs P1-P2 are produced by ribosomal frameshift, acting as RNA-dependent RNA polymerase. ORFs 3, 4, and 5 are translated from sub-genomic RNAs, encoding P3, P4, and P3-P5 proteins, respectively. P3 is a coat protein (CP), P4 is a movement protein (MP), and P3-P5 is involved in transmission by aphids, acting as a readthrough protein [11].

The analysis of the full-length genome sequences of 33 CABYV isolates showed that they formed several distinct phylogenetic groups, which were associated with different geographic regions and collection time periods [12]. A study on the genetic diversity and evolutionary history of CABYV and closely related viruses used phylogenetic and molecular clock analyses based on partial sequences of the CP gene of 173 isolates from 19 countries across six continents. The study found that CABYV is a monophyletic group that originated in the Old World, with a putative ancestral host in the genus *Cucumis* [13]. Subsequently, based on the sequencing and phylogenetic studies of different isolates, CABYV was classified into four main groups: Asian or Chinese (C), Mediterranean (N), Taiwanese (TW), and recombinant groups (R) [5]. The genetic diversity and phylogeny of CABYV were analyzed based on full genome sequence of 18 isolates collected from various regions worldwide in another study. The analysis showed that the CABYV isolates formed two distinct phylogenetic clusters, with one cluster containing isolates from Africa and Asia, while the other cluster comprising isolates from Europe. Additionally, several recombination events were identified within the CABYV genome, which may have contributed to the emergence and diversification of new strains of the virus [14]. High-throughput sequencing was used to analyze the genetic diversity of CABYV isolates collected from Spain and other European countries. The analysis revealed four main phylogenetic groups of CABYV isolates, which were further subdivided into several subgroups [15]. A recent study on the genetic diversity and geographic distribution of CABYV and other cucurbit-infecting viruses in Israel, using a combination of molecular and serological techniques, found that CABYV is the most prevalent virus infecting cucurbit crops in Israel, with a prevalence rate of 46%. The study also identified several genetically distinct strains of CABYV, associated with different geographic regions and host plants in Spain [15]. 

Next-generation sequencing (NGS) was used for the first time to detect CABYV in melons showing yellow symptom in South Korea [13]. CABYV has been reported to cause melon disease in Europe, Africa, and America. It can also display simultaneous infection with Potyviruses in pumpkins [16]. CABYV has also been reported in cucurbits, including melons, cucumbers, squash, and watermelons in the primary production regions in Iran [17]. According to the research, CABYV is one of the most prevalent viruses in cucumber greenhouses in Tehran and Alborz provinces. The phylogenetic evaluation of CABYV-C isolates from the cucumber cultivation regions of Iran based on previous research and RdRP gene sequence analysis revealed the presence of at least two CABYV-C subpopulations in these areas [17]. However, there is limited information on CABYV isolates’ exact phylogenetic position or genetic diversity based on the CP gene in the Zanjan region. 

Understanding the genetic diversity and distribution of plant viruses is crucial for developing effective disease management strategies and ensuring global food security [18,19]. The molecular characterization of coat protein genome sequences and comparing genetic variability sampled from different parts of Iran with the isolates from NCBI possibly shed light on the dark part of the information regarding CABYV. The identification of distinct phylogenetic clusters and genetic recombination events within the CABYV genome might provide valuable information for the development of strategies to control the spread of this emerging viral pathogen of cucurbit crops.

## 2. Materials and Methods

### 2.1. Sampling

A total of 200 samples were collected from most cucurbit growing areas in west and northwest of Iran, including Zanjan, Hamedan, Kurdistan, Ardabil, North Azerbaijan, and West Azerbaijan provinces. These samples were collected during the growing season of 2019–2023 from cucurbit, cucumber, squash, and melon plants which showed leaf yellowing and thickening symptoms. Each sample was assigned a unique number based on the time and date of collection and immediately frozen at −80 °C for subsequent total RNA extraction.

### 2.2. RNA Extraction, RT-PCR

The suspected samples exhibiting viral symptoms were subjected to RNA extraction using a modification of the CTAB method as described before [20]. The extracted total RNA was then used to synthesize cDNA, employing a kit with a random hexamer primer, following the manufacturer’s instructions. Subsequently, a pair of specific primers corresponding to the complete sequence of CABYV coat protein gene (P3) were employed to perform the PCR amplification of an expected DNA fragment of approximately 600 bp. The primer sequences used were CP5-5′-ATGAATACGGCCGCGGCTAGAAATC-3′ and 5′-CTATTTCGGGTTCTGGACCTGGCA-3′ [21]. The PCR program was optimized with the following conditions: initial denaturation at 94 °C for 2 min, followed by 35 cycles of 94 °C for 30 s, 64 °C for 30 s, and 72 °C for 30 s. The final polymerization step was set at 72 °C for 5 min. Selected amplified fragments were sent for sequencing to BMG Company.

### 2.3. Recombination Analysis

In May 2023, all 76 isolates with complete genome sequences available in the NCBI GenBank were retrieved and then aligned using the ClustalW algorithm in the MEGA X program. The 5′ and 3′-untranslated regions were removed to create the “complete genome” alignment, which had a length of approximately 5690 base pairs. The complete coat protein sequences of 76 GenBank isolates, as well as new isolates obtained in this study, were added to the ORF3/CP alignment (Table 1). We used the Recombination Detection Program 4 (RDP4) with its full suite of options, including Siscan, RDP, MaxChi, Chimaera, Bootscan, 3Seq, and GENECONV, with default parameters and a *p* value threshold of 0.05, to scan for any phylogenetic anomalies in the alignments. Anomalies detected by fewer than five algorithms were disregarded. The parent and recombinant relationship of the two recombinant strain clusters were confirmed by separating the alignments into regions that did, or did not, contain the recombinant regions, and comparing trees generated from those part sequences.

### 2.4. Phylogenetic Analysis

The identity analysis of complete CABYV sequences in GenBank databases from around the world were conducted using NCBI-BLAST (http://www.ncbi.nlm.nih.gov/BLAST/ (accessed on 11 May 2023)). Sequence alignment was performed with MEGA X v.10.2.4 software [22] and the Clustal W program [23]. Additionally, new sequences obtained during this research were compared to those deposited in GenBank up to 2023 (Table 1). A multiple sequence alignment of the retrieved and new 18 Iranian sequences of the complete P3 (CP) gene was performed using the Clustal W algorithm in MEGA X software. To generate phylogenetic tree-based overlapping coat protein and movement protein ORFs (ORFs3 and 4), recombinant groups and some other isolates belong to different groups that have a <0.5% genetic distance were omitted.

The evolutionary relationships and phylogenetic tree of all CABYV isolates were generated using the maximum likelihood (ML) method based on Tamura 3-parameter’s model [24] with gamma-distributed (G) and invariant sites (I) (T92 + G + I) and codon positions (1st + 2nd + 3rd + noncoding sites), as implemented in MEGA X software v.10.2.4. The branch support was calculated using the bootstrap method based on 1000 replications, and all branches with less than 50% bootstrap were removed [22]. The pairwise nucleotide sequence identity matrix was generated using the Sequence Demarcation Tool (SDTv1.2) [25].

**Table 1:** List of the origins, hosts, and accession numbers of CABYV isolates/strains whose complete genome and ORF3 (P3, CP) sequences were used in the present study.

Country	Isolates/strains	Host	Accession Number	Collection Date
South Korea (30)	SW1, SW2, CY3, C-HS1, WM-YS10, NW2, GS2, CY6, NW5, GM16, HS1,GS6, HD118, HD1, GS1, SW25, SW1(14), HS2, NW18, GM7, NW1, NW2(14), SW64, CY4,melon-GJ, Cumber-AS-kr1, M-CY31, M-BY1, C-AS1, K1	melon, melon, melon, cucumber, watermelon, melon, melon, melon, melon, melon, melon, melon, melon, melon, melon, melon, melon, melon, melon, melon, melon, melon, melon, melon, melon, cucumber, melon, melon, cucumber, melon	KR231959, KR231961, KR231942, MG257900, MG257903, KR231955, KR231948, KR231944, KR231957, KR231946, KR231952, KR231949, KR231951, KR231950, KR231947, KR231962, KR231960, KR231953, KR231958, KR231945, KR231954, KR231956, KR231963, KR231943, MW509732, MZ508305, MG257902, MG257901, MG257899, LC082306	2014, 2014, 2013, 2016, 2016, 2013, 2014, 2014, 2013, 2013, 2014, 2014, 2014,2014, 2014, 2014, 2014, 2014, 2014, 2013, 2014, 2014, 2014, 2013, 2020, 2021, 2016, 2016, 2016, 2014
Japan: Okayama (1)	CABYV-JAN	Cucumber	GQ221224	-
China (5)	CABYV-FJ, -, -, CABYV-CZ, CABYV-QY	Squash, cantaloupe, cucurbit, zucchini, suakwa vegetable sponge	GQ221223, EU636992, EU000535, HQ439023, MT943520	-, -, -, -, 2017
USA (1)	BL-4	Pumpkin	MK055337	2017
Spain (29)	Sq/2004/1.9, Sq/2003/7.2, MEC-12, LP63, 64.2M/M, 9.2Z/A, 9.1M/M, 9.1M/M, 8.1M/M, 8.1Me/A, 7.1M/M, 67.1Z/M, 59.1M/A, 53.1M/M, 3.2M/M, 23.1M/M, 22.1Z/M, 2.1Z/M, 2.1M/M, 2.1M/M, 19.1Z/A, 19.1M/M, 19.1M/M, 18.1Z/A, 18.1M/A, 11.1Z/M, 10.1Z/M, 1.1M/M, Sq/2005/9.2	-, -, melon, watermelon, melon, zucchini, melon, melon, melon, melon, melon, zucchini, melon, melon, melon, melon, zucchini, zucchini, melon, melon, zucchini, melon, melon, zucchini, melon, zucchini, zucchini, melon, -	JF939814, JF939812, MW051362, MW051363, OM948857, OM948856, OM948855, OM948854, OM948853, OM948852, OM948851, OM948850, OM948849, OM948848, OM948847, OM948846, OM948845, OM948844, OM948843, OM948842, OM948841, OM948840, OM948839, OM948838, OM948837, OM948836, OM948835, OM948834, JF939813	2017, -, -, 2014, 2019, 2018, 2013, 2019, 2014, 2016, 2014, 2013, 2018, 2020, 2020, 2017, 2015, 2019, 2015, 2013, 2011, 2019, 2019, 2015, 2015, 2016, 2012, 2020, 2014, -
Papua New Guinea (1)	10PN	cucumber	MG780352	2016
Taiwan (2)	CABYV-R-TW82, CABYV-C-TW20	*Luffa aegyptiaca*, *Momordica charantia*	JQ700306, JQ700305	2009, 2008
France (1)	EM160093	melon	MT027103	2016
Indonesia (1)	-	cucumber	LC472499	2017
India (2)	POL-WM, POL-SQ	watermelon, squash	MN688220, MN688219	

### 2.5. Population Genetic Parameters

This study utilized the DnaSP version 6.10.01 program [26] to examine the genetic differentiation parameters of the CP gene among various populations. These parameters included haplotype diversity (*Hd*), the number of polymorphic sites (S), the total number of mutations (η), the average number of nucleotide differences (k), average pairwise nucleotide diversity (π = Pi), and ω (dN/dS). The neutral selection hypothesis was also investigated using Tajima’s *D* [27] and Fu and Li’s D* and F* [28] statistical tests. The program revealed that the genes were under one of three types of selection pressures: negative (purifying), neutral, and positive (diversifying) selection when the dN/dS ratio was <1, =1, and >1, respectively [26]. Additionally, population differentiation tests, such as *K*_S_*, Z*, *S*_nn_, and *F*_ST_ (fixation index), were conducted to estimate inter-population diversity with 1000 replicates [29]. The *F*_ST_ value of higher than 0.33 suggests limited gene flow and further genetic isolation among tested populations [30]. The frequency graphs of pair-wise differences among populations were also analyzed using the population size changes option in DnaSP software. The study further employed the single-likelihood ancestor counting algorithm (SLAC) in the MEGA X software and Datamonkey webserver (https://datamonkey.org (accessed on 19 June 2023)) to evaluate dS, dN, identifying codons, and clades under natural selection.

### 2.6. Molecular Dating Analysis

The study established the congruence between the lineages of Poleroviruses and their hosts. The divergence times of the CABYV population, along with eight other Poleroviruses, known to have a close genetic relationship, were determined. The six other Poleroviruses were chickpea chlorotic stunt virus (CpCSV), faba bean polerovirus 1 (FBPV-1), pepo aphid-borne yellows virus (PABYV), melon aphid-borne yellows virus (MABYV), Pumpkin polerovirus, suakwa aphid-borne yellows virus (SABYV), and a *Polemovirus* species, namley poinsettia latent virus (PnLV), which was used as an outgroup. The age of internal nodes was evaluated to estimate these divergence times, and the TimeTree was reconstructed using the fast-dating RelTime-ML computational method under the Tamura 3-parameter model [24], which was implemented in MEGA X software [22]. The default calibration of time to most recent common ancestors (TMRCA) was utilized [31]. A discrete gamma distribution was used to model evolutionary rate differences among sites (5 categories (+G, parameter = 2.2907)). The rate variation model allowed for some sites to be evolutionarily invariable ([+I], 8.65% sites). This analysis involved 25 nucleotide sequences. Codon positions included were 1st + 2nd + 3rd + noncoding. There was a total of 6352 positions in the final dataset. Times were not estimated for outgroup nodes because the RelTime method uses evolutionary rates from the ingroup to calculate divergence times and does not assume that evolutionary rates in the ingroup clade apply to the outgroup. All divergence times shown are relative times as no calibrations were provided.

## 3. Results

### 3.1. Field Observation

During the cucurbit-growing season in Iran, many regions in the west and northwest of Iran, including the Zanjan, Hamedan, Kurdistan, Ardabil, North Azerbaijan, and West Azerbaijan provinces, were visited, and various symptoms consistent with those caused by CABYV infection were observed in the cultivated cucurbits, including yellowing, leaf deformation, mottling, vein clearing, and reduced yield. Melons in different regions showed stunting as well as the additional symptoms mentioned previously. On ridge gourd (*Luffa acutangula*), interveinal yellowing was common, while bitter gourd (*Momordica charantia*) displayed yellowing with green vein-banding or yellow mosaic pattern, and watermelon mostly exhibited yellow mosaic pattern. Melons typically exhibited interveinal chlorosis or yellow mosaic pattern. These symptoms were often similar to nutrient deficiencies, and various factors such as environmental conditions, virus strain, and plant variety can also affect their expression. Some cucurbits infected by a virus may not display any symptoms at all.

**Figure 1:**
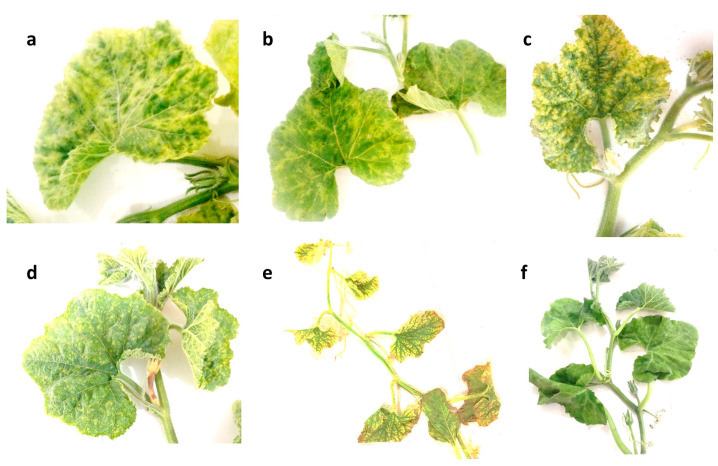
The most symptoms that observed in the visited fields and represent positive infection by CABYV. (**a**) Yellowing, (**b**) mild mosaic pattern, (**c**) yellowing and mosaic pattern, (**d**) mild mosaic pattern, (**e**) severe yellowing, (**f**) mild mosaic pattern.

In field observations, most samples showed systemic symptoms, including the yellowing of leaves and fruits, thickening and brittle leaves, the curling or rolling of leaves, abnormal growth in leaves and stems, mild to severe mosaic pattern, green stunting, leaf distortion and asymmetry, stunted plant growth, and vein distortion. Severe symptoms can be observed in the first half of fall, although they seem to often be confused with physiological changes. The areas where most CABYV-positive samples were found was in the west of Iran. However, the specific symptoms of CABYV vary depending on the type and variety of cucurbit, as shown in Figure 1.

Throughout the growing season, from spring to fall, different symptoms were noticeable, with severe symptoms being observed in the late spring and early summer. As temperatures increased in the summer, the severe symptoms changed to mild symptoms from late summer to early fall. This change could be attributed to a decrease in virus titer due to high temperatures, which is common in many cucurbits’ viruses. CABYV infection on *Cucurbitaceae* usually causes symptoms ranging from chlorosis to yellowing, with green venial patterns, yellow mosaic pattern, and the thickening of older leaves.

### 3.2. PCR Amplification

PCR, using the specific primers, successfully amplified a 600 bp fragment of CABYV genome, including the full CP gene, in 38 samples. The infected melons exhibited yellowing symptoms, while bitter gourds showed yellow mosaic symptoms (Figure 1). Eighteen positive samples were sequenced and then were assigned accession numbers OQ351953 to OQ351959, and OQ703990 to OQ704000 after being deposited in the NCBI GenBank.

### 3.3. Recombination Isolates

The RDP analysis of CABYV alignment showed that around 40% of tested isolates (27 isolates) were recombinants originating from single recombination events (Table S1). Using these methods, a total of five confirmed recombination events were identified among 76 virus isolates from South Korea, the USA, China, India, and Spain. All recombination events met the triple criterion for acceptance: detection by more than four methods, consensus score in RDP4 > 40, and *p* < 0.01. The recombinant sequences were excluded from the alignment used for phylogenetic and population genetic analysis because they were able to distort the results of most algorithms used for reconstructing phylogenies. The CP gene alignment were also analyzed using RDP4, but no additional recombinant sequences were identified.

### 3.4. Phylogenetic Analysis

The analysis of new Iranian sequences revealed that they belong to the complete ORF3 and partial ORF4 of CABYV genome, with an encoded length of approximately 199 amino acids and varying similarities. Upon aligning the nucleotide sequences, it was observed that the identities between the new Iranian isolates and other tested isolates ranged from 94.3% to 99.3%, with the most significant differences observed in isolates from Thailand (KF815686, KF815687), USA (MK055337), South Korea (MZ366313, MZ508305, KR231948), Japan (GQ221224), and China (EU000535). The obtained sequences from CP genes in this study showed a high sequence identity (94.8% to 99.5%) among themselves.

Additionally, the alignment of deduced amino acid sequences indicated that the identities between the Iranian isolates and other isolates ranged from 96.2% to 99.5%, with the most amino acid differences observed in isolates from Thailand and South Korea. The pairwise nucleotide sequence identity matrix is presented as a matrix. The sequencing analysis results revealed that fragments ORF3 and ORF4 accounted for approximately 21% of the complete genome of CABYV.

**Figure 2:**
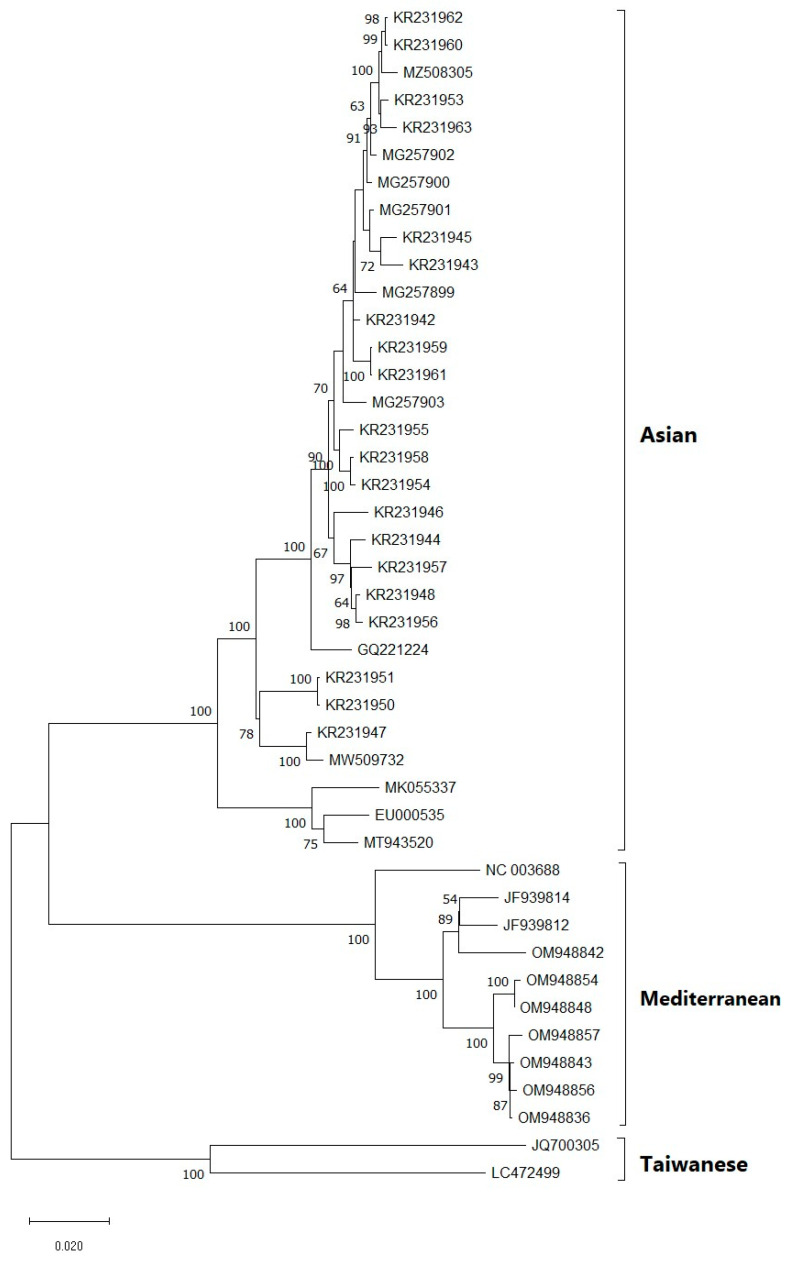
Phylogenetic tree of CABYV isolates/strains reconstructed based on complete genome sequences via the maximum likelihood (ML) method based on Tamura’s three-parameter model with gamma-distributed (G) and invariant sites (I) (T92 + G + I) in MEGA X. Bootstrap values over 50% are given at the nodes.

**Figure 3:**
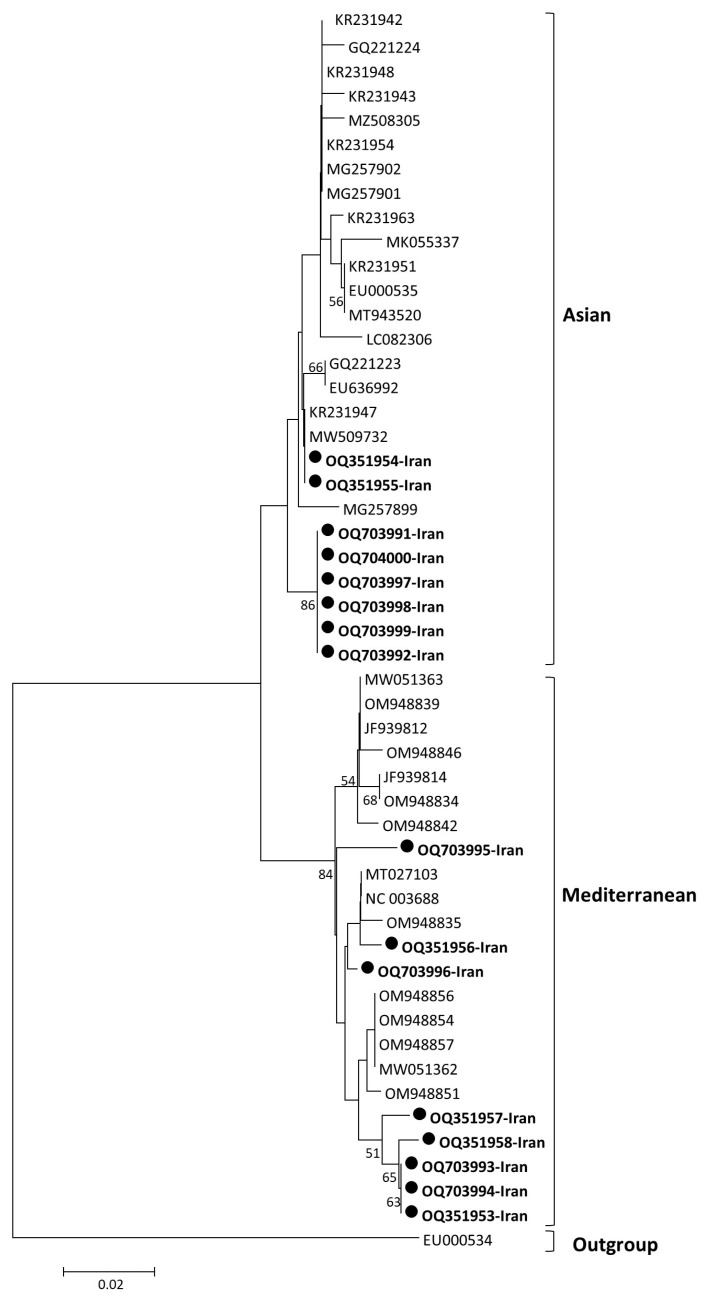
Phylogenetic tree of CABYC isolates/strains reconstructed based on complete ORF3/ORF4 sequences via the maximum likelihood (ML) method based on Tamura’s three-parameter model with gamma-distributed (G) and invariant sites (I) (T92 + G + I) in MEGA X. Bootstrap values over 50% are given at the nodes. The new Iranian isolates are shown with black circles and in bold.

Phylogenetic trees generated using different models in the NJ method displayed similar topologies, identifying two major clades based on the complete genome (Figure 2) and ORF3/4 (Figure 3), which separated the population into Asian and Mediterranean groups. Furthermore, a recombinant group was identified in the phylogenetic tree based on the complete genome, which included sequences excluded in the CP phylogenetic tree. The Asian group comprised isolates from South Korea, Japan, and China, while the Mediterranean group were predominantly from Spain. The recombinant group consisted of isolates from Taiwan (JQ700306) and Indonesia (LC472499). After excluding the recombinant group and some isolates with less than 0.5% genetic distance, a phylogenetic tree was constructed based on the overlapping coat protein and movement protein ORFs (ORF3/4), which also showed two main groups (Asian and Mediterranean). These groups were consistent with the phylogenetic tree based on the complete genome. The study analyzed 18 new isolates from Iran, which were grouped into the Asian and Mediterranean groups. Two isolates (OQ351954, OQ351955) were closely related to isolates from China and South Korea, while six isolates (OQ703991, OQ703992, OQ703997, OQ703998, OQ703999, and OQ704000) formed an independent subcluster within the Asian group. Eight new isolates in the Mediterranean group were grouped into different subclusters, including a distinct group consists of isolates from cucumber and pumpkin, namely OQ351953, OQ351957, OQ351959, OQ703993, and OQ703994, and a subcluster consisted of OQ703996 and OQ351956 with isolates from Spain and France (Figure 3). Additionally, one of the new isolates from Iran in the Mediterranean group was genetically distinct from the other isolates. Two new isolates that showed recombinant events: OQ703990 and OQ351958 were omitted from the ORF3/4 phylogenetic tree. Interestingly, the host species did not appear to be correlated with the different clades in the phylogenetic trees, suggesting that geographical regions may have a stronger influence on the grouping of isolates based on genetic analysis. Furthermore, the subgrouping of the two main groups (Asian and Mediterranean) based on the complete genome and ORF3/4 was consistent. These results also confirmed that the primer pairs used in RT-PCR are able to amplify the ORF3/4 regions of both Asian and Mediterranean strains.

### 3.5. Genetic Variation and Selection Pressure Analyses

The genetic diversity of CABYV populations was examined using various parameters, including nucleotide diversity (π), haplotype diversity (Hd), segregating sites (S), and the average number of nucleotide differences per site (η) for different regions of the virus genome (ORF0, ORF1 + 2, ORF3, ORF4, and ORF3 + 5) and new isolates from Iran. The highest nucleotide diversity was observed in ORF0, ORF3 + 5, and the complete genome of CBYV, with π values of 0.06814, 0.06633, and 0.06459, respectively. The π values for Asian and Mediterranean groups based on the complete genome and ORF3 (P3, CP) were <0.3, with the lowest values obtained from the ORF3 (P3, CP) analysis (0.01115 and 0.01432). The results also indicated that ORF3 (P3, CP) was the most conserved region, with the lowest Hd, S, η, and k values among all the ORFs analyzed. Moreover, the analysis of 44 and 50 isolates’ complete genome and ORF3 (P3, CP) sequences revealed 43 and 26 distinct haplotypes, respectively, indicating the presence of genetic diversity in CBYV populations (Table 2). These findings can help in the management and control of CBYV spread.

### 3.6. Neutrality Test

**Figure 4:**
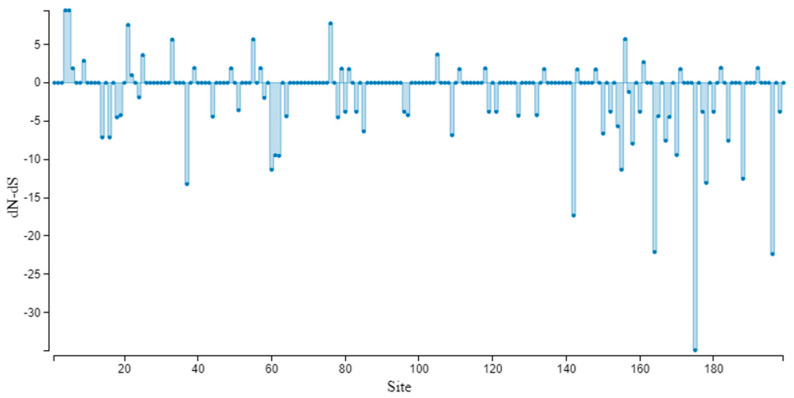
SLAC site graph to positively and negatively identify codons/sites in terms of ORF3 (P3, CP).

**Figure 5:**
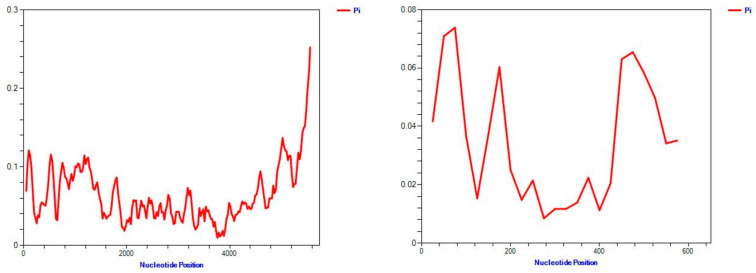
Sliding-window plot of polymorphism and divergence levels of CABYV isolates based complete genome (**left**) and ORF3 (P3, CP) (**right**).

Based on the analysis of CABYV populations using complete genome, ORFs, and phylogroups, the dN/dS (ω) ratio was estimated to be less than 1. The ORF0 analysis revealed the highest ω value of 0.52, while the complete genome and ORF3 (P3, CP) showed ω values of 0.4294 and 0.2855, respectively. The dN/dS (ω) values for Asian and Mediterranean groups based on ORF3 (P3, CP) were 0.1733 and 0.4606, respectively (Table 2). Overall, the results indicate that negative selection is operating on all genes and clades of CABYV populations. The SLAC method for ORF3 (P3, CP) genes indicated that 25 sites are under positive selection and other sites under negative selection with a *p*-value threshold of (*p* ≤ 0.1) (Figure 4). The population expansion was analyzed using mismatch distribution graphs, which demonstrated multimodal curves (Figure 5). These findings suggest that CABYV populations are subjected to strong negative evolutionary constraints across complete genome, ORFs, and phylogroups. In addition, the sliding-window plot of polymorphism and the divergence levels of CABYV isolates for complete genome and ORF3 showed different polymorphism at the sites (Figure 5).

The molecular variation patterns in the segregated sites of CABYV populations were analyzed using Tajima’s *D* and Fu and Li’s D* and F* statistical tests. The results of the molecular variation analysis suggest that non-significantly positive values were obtained for the complete genomes, ORFs, and phylogroups in all of the statistical tests. For the French population, significantly negative values were calculated for the MP gene in all three statistical tests and only for Tajima’s D test for the CP gene. Among all the analyses, non-significantly negative values were obtained for all complete genomes, ORFs, and phylogroups except for the Mediterranean population, which showed non-significantly positive values for the complete genome in all three statistical tests (Table 3).

### 3.7. Genetic Differentiation

The results of the genetic differentiation analysis showed two distinct clades (Asian and Mediterranean) with significant *K*_S_* and Z* independent tests. However, the *S*_nn_ value was not significant between the Asian and Mediterranean clades based on both the complete genome and ORF3 (P3, CP). The *F*_ST_ values between the two clades (Asian and Mediterranean) were estimated to be 0.758 and 0.718 (>0.33) based on the complete genome and ORF3 (P3, CP), respectively, indicating a high level of genetic differentiation between these two clades (Table 4). Furthermore, based on the *S*_nn_ value of 1.000, the two clades of CABYC population, as defined by the complete genome and ORF3 (P3, CP), were distinct from other populations around the world (Table 4).

### 3.8. Divergence Time Analysis

**Figure 6:**
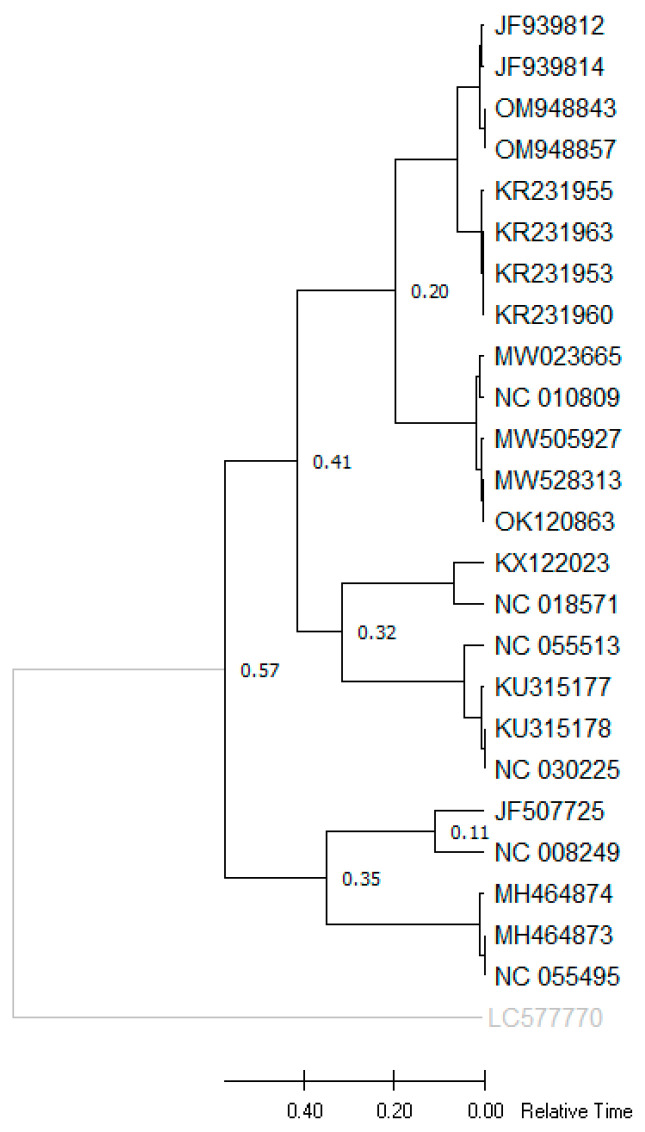
TimeTree analysis for nine *Polerovirus* members using the RelTime method. A TimeTree inferred by applying the RelTime method to the user-supplied phylogenetic tree whose branch lengths were calculated using the maximum likelihood (ML) method and Tamura’s three-parameter substitution model. The estimated log likelihood value of the tree is −58180.90. A discrete gamma distribution was used to model evolutionary rate differences among sites (five categories (+G, parameter = 2.2907)). The rate variation model allowed for some sites to be evolutionarily invariable ([+I], 8.65% sites). This analysis involved 25 nucleotide sequences. Codon positions included were 1st + 2nd + 3rd + noncoding. There was a total of 6352 positions in the final dataset. Evolutionary analyses were conducted in MEGA X. Chickpea chlorotic stunt virus (CpCSV), faba bean polerovirus 1 (FBPV-1), pepo aphid-borne yellows virus (PABYV), melon aphid-borne yellows virus (MABYV), pumpkin polerovirus, and suakwa aphid-borne yellows virus (SABYV) were included in the analysis. A *Sobemovirus* species, namely poinsettia latent virus (PnLV), was used as an outgroup.

Based on the obtained phylogenetic TimeTree, the species of *Polerovirus* were grouped into two main clusters, with one cluster encompassing CABYV, MABYV, Pumpkin polerovirus, SABYV, and PABYV, and with CpCSV and FBPV-1 grouped in another cluster. As shown in Figure 6 and Table 5, two species of this genus, i.e., CABYV and MABYV, have a close phylogenetic relationship, and our molecular clock estimation was obtained 0.20 Ma between two these species. This method (i.e., the estimation of divergence time) can be particularly useful for species lacking fossil histories. PnLV sequence from *Polemovirus* genus was selected as an outgroup.

## 4. Discussion

Some viral symptoms, including vein clearing, yellowing, and mosaic pattern, were observed extensively in a wide range of plants in many different regions. Generally, the symptoms observed in pumpkin, zucchini, and melon were much more pronounced, severe, and clearer than in other hosts. However, mosaic symptoms with yellowing were observed to be less severe in pumpkin than in other cucurbits in the fields. Vein clearing, which often leads to leaf distortion, was observed more often in cucumber than in other hosts and was even absent in pumpkin and sweet pumpkin plants. Overall, the lowest and mildest severities were observed in cucumber plants, possibly indicating the sensitivity and resistance of plant cultivars or virus host restriction. This study presented a unique occurrence in which both Asian and Mediterranean strains of CABYV were found in a same country, and have a relatively similar incidence, as indicated in the phylogenetic trees of 16 selected Iranian isolates. Some regions had a higher prevalence of these symptoms, probably due to a particular variant of the virus dominating in that area. However, symptom expressions caused by both strains on cucurbits were observed to be similar and indistinguishable in Iranian fields. Likewise, the Asian isolates were reported to induce yellowing and mosaic symptoms in melon cultivated in South Korea [13] while the Mediterranean isolates caused the variations of yellowing on watermelon leaves in Spain [5].

Viral symptoms were variable in different growth stages, from early to mid-summer and from late summer to early fall. Viral symptoms were more frequently observed in late growth stages, which may be due to the higher likelihood of mixed infection and the presence of multiple viruses in samples collected at the end of the growth season. Vectors actively transmitting the virus in autumn, a season with a relatively low temperature, could be another reason for infection in the late growth season leading to the highest incidence. In the spring, viral symptoms were already noticeable in pumpkin farms. In the summer, the lowest and weakest symptoms begin to show, while in the autumn, viral symptoms were severe and had a high percentage of occurrence, indicating that viral symptoms were milder and even disappeared under high temperature conditions.

In order to identify the occurrence of viral CABYV infection in cucurbit crops, population analysis based on coat protein can play a prominent role. Recombination and mutation can affect the evolution of plant virus populations, therefore identifying haplotypes and relationships between CABYV populations based on the CP can shed light on population evolution. The genetic diversity of CABYV populations may be influenced by specific hosts and/or geographic origin [19,32]. In this study, we analyzed the genetic structure of a CABYV population under special circumstances.

An observation on CABYV isolates collected in Murcia and Castilla-La Mancha, Spain, identified three codons in the ORF2 of *Ecballium elaterium* (a wild cucurbit) to be under positive selection but not in the melon population. Furthermore, the *F*_ST_ values among *E*. *elaterium* and melon subpopulations from different fields ranged from 0.35 to 0.61, suggesting that the genetic differentiation of the isolates could be attributed to host adaptation [33]. Our phylogenetic analysis of the isolates, based on the complete genome, revealed three main clades, including Asian, Mediterranean, and R-groups. The largest clade, Asian, contained isolates from South Korea, Japan, and China (Figure 2). However, according to the phylogenetic tree of the CP gene, our isolates belonged to two clades, Asian and Mediterranean (Figure 3). Among all isolates from different regions, the isolates from China and South Korea were closely related to two isolates from our study (OQ351954 and OQ351955), indicating the effect of geographic origin on CABYV genetics. 

The CP gene (ORF3) showed lower genetic variation in all parameters, such as the number of mutations and π value. The CP region is known as the most conserved region among all ORFs, and therefore, this analysis demonstrated the capability of CP sequences to play a main role in resolving CABYV phylogeny (Table 2). Although both Asian and Mediterranean clades showed low π values (<0.3), the Mediterranean clade showed greater nucleotide diversity than the Asian clades based on the complete genome (Table 2). Our analysis showed that *Hd* was 0.960–1.000, indicating high levels of haplotype diversity in this study. Overall, ω was estimated to be <1 for CABYV populations, which suggests that all genes and clades of CABYV populations are under negative selection (Table 2). However, the most significant differences were observed between the new Iranian isolates and isolates from Thailand, China, South Korea, Japan, and the USA. Based on the results of gene flow analysis, two clades (Asian and Mediterranean) exhibit high genetic differentiation. The *F*_ST_ values for both the complete genome and CP (0.75874 and 0.71847, respectively) indicate genetic separation between the Asian and Mediterranean clades. The *S*_nn_ value (1.000) further confirms that the two clades are distinct from other populations around the world (Table 3). Using fast-dating RelTime-ML, a TimeTree was reconstructed, and according to divergence times of the CABYV population, eight poleroviruses showed a close genetic relationship with CABYV.

## 5. Conclusions

In conclusion, understanding the genetic diversity of viruses is crucial for developing strategies for virus control. Therefore, in this study, we analyzed the CABYV population and genetic diversity. Our research aimed to reveal patterns of population genetic structure of CABYV in this region. Based on previous studies and our analysis, we confirmed the existence of Asian and Mediterranean clades in this region, and we identified three phylogroups based on the complete genome.

## Figures and Tables

**Table 2:** Genetic diversity and polymorphism analyses of complete genome, ORF0, ORF1 + 2, ORF3 (CP), ORF4 (MP), and ORF3 + 5 of CABYV from different phylogroups and regions.

Population	*N*	*h*	*Hd*	*S*	η	*k*	π	dS	dN	ω
Complete genome	44	43	0.999	1575	1889	359.165	0.06459	0.14167	0.04046	0.2855
Phylogroups (Complete Genome)										
Asian	31	31	1.000	802	843	142.168	0.02505	0.05880	0.01464	0.2489
Mediterranean	11	10	0.982	470	488	162.145	0.02860	0.07279	0.01495	0.2053
ORF0	44	38	0.990	216	247	48.854	0.06814	0.10700	0.05564	0.5200
ORF1 + 2	44	43	0.999	860	1000	187.080	0.05920	0.10641	0.04486	0.4215
ORF4	50	26	0.960	24	25	6.167	0.02909	0.06467	0.01814	0.2805
ORF3 + 5	44	42	0.998	573	710	132.185	0.06633	0.19262	0.02610	0.1354
ORF3 (CP)	50	26	0.960	24	25	6.167	0.02909	0.04990	0.02143	0.4294
Phylogroups (new isolates) (Coat Protein)										
Asian	27	12	0.900	13	13	2.365	0.01115	0.03583	0.00621	0.1733
Mediterranean	23	14	0.945	12	12	3.036	0.01432	0.05017	0.02311	0.4606

*N*: number of isolates, *h*: number of haplotypes, *Hd*: haplotype diversity, *S*: number of variable sites, η: total number of mutations, *k*: average number of nucleotide differences between sequences, π: nucleotide diversity (per site), dN: non-synonymous nucleotide diversity, dS: synonymous nucleotide diversity, ω: dN/dS.

**Table 3:** Results from demography test statistics between sequences of complete genome, ORF0, ORF1 + 2, ORF3 (CP), ORF4 (MP), and ORF3 + 5 of CABYV populations.

Population	Fu and Li’s D*	Fu and Li’s F*	Tajima’s *D*
Complete genome	−0.85487 ns	−0.92764 ns	−0.64287 ns
Phylogroup			
Asian	−1.10647 ns	−1.37784 ns	−1.26911 ns
Mediterranean	0.31512 ns	0.22766 ns	−0.13000 ns
ORF3 (CP)	−1.02637 ns	−0.64835 ns	0.34283 ns
Phylogroup			
Asian	−1.49049 ns	−1.57070 ns	−1.00962 ns
Mediterranean	−0.25873 ns	−0.29129 ns	−0.22998 ns
ORF4 (MP)	−1.02637 ns	−0.64835 ns	0.34283 ns
ORF0	−0.84544 ns	−0.86372 ns	−0.51353 ns
ORF1 + 2	−0.83674 ns	−0.93552 ns	−0.69127 ns
ORF3 + 5	−0.95533 ns	−1.03074 ns	−0.70499 ns

**Table 4:** Genetic differentiation estimates for lineages of CABYV, based on complete genome and CP gene sequence comparisons.

Comparison	^α^*K*_S_*	^α^*K*_ST_*	*p* Value	^α^Z*	*p* Value	*S*_nn_	*p* Value	^β^ *F*_ST_
Complete genome								
Phylogroup								
All (n = 44)/Asian (n = 31)	5.1803	0.0002	0.422 ns	6.9397	0.633 ns	0.1733	1.000 ns	0.104
All (n = 44)/Mediterranean (n = 11)	5.3621	0.0423	0.000 ***	6.1228	0.000 ***	0.6242	0.774 ns	0.482
Asian (n = 31)/Mediterranean (n = 11)	4.7411	0.1243	0.000 ***	5.2430	0.000 ***	1.000	0.000 ***	0.758
ORF4 (CP)								
Phylogroup								
All (n = 50)/Asian (n = 27)	1.5485	0.0566	0.000 ***	6.9248	0.000 ***	0.5202	0.656 ns	0.243
All (n = 50)/Mediterranean (n = 23)	1.6342	0.0718	0.000 ***	6.7496	0.000 ***	0.5191	0.815 ns	0.293
Asian (n = 27)/Mediterranean (n = 23)	1.1987	0.3256	0.000 ***	5.4576	0.000 ***	1.000	0.000 ***	0.718

*** *p* value < 0.001; ^α^*K*_S_*, ^α^*K*_ST_*, ^α^Z* and *S*_nn_ are test statistics of genetic differentiation; ^β^*F*_ST_, coefficient of gene differentiation, which measures inter-population diversity.

**Table 5:** Estimated CABYV TMRCA based on the ratio of the patristic distances within the complete genome sequence maximum likelihood tree of the eight poleroviruses.

Species	Mean Patristic Distance	Ratios of CABYV and Other Patristic Distances
CABYV	0.20	1
MABYV	0.20	1
SABYV	0.32	1.6
Pumpkin polerovirus	0.32	1.6
PABYV	0.32	1.6
CpCSV	0.35	1.75
FBPV-1	0.35	1.75

## Data Availability

Sequences of 18 novel Iranian CABYV isolates have been made available in NCBI GenBank, reference numbers OQ351953 to OQ351959 and OQ703990 to OQ704000.

## Supplementary Materials

The following supporting information can be downloaded at: https://www.mdpi.com/article/10.3390/v15081714/s1, Supplementary Table S1: Putative recombination events detected in the ORF3/4 of CABYV genome via RDP4 analysis.
