# Supplementary material for: Genetic Variation between Asian and Mediterranean Populations of Cucurbit Aphid-Borne Yellows Virus
Source: Viruses. 2023 Aug 9;15(8):1714. doi: 10.3390/v15081714 (PMC10457933; doi:10.3390/v15081714)
Supplement: Supplementary file 1 [file viruses-15-01714-s001.zip › viruses-2498128-supplementary.pdf]

Genetic Variation between Asian and Mediterranean Populations of Cucurbit Aphid-Borne Yellows Virus

Parastoo Pouraziz [1], Milad Yousefi [1], Adyatma Irawan Santosa [2,*] and Davoud Koolivand [1]

[1] Department of Plant Protection, Faculty of Agriculture, University of Zanjan, Zanjan 45371-38111, Iran; koolivand@znu.ac.ir (D.K.)
[2] Department of Plant Protection, Faculty of Agriculture, Universitas Gadjah Mada, Yogyakarta 55281, Indonesia
* Correspondence: adyatma.i.s@ugm.ac.id

Table S1. Putative recombination events detected in the ORF3/4 of CABYV genome by RDP4 analysis.

No.	Recombinant Sequence(s)	Minor Parental Sequence(s)	Major Parental Sequence(s)	RDP	GENECONV	Bootscan	Maxchi	Chimaera	SiSscan	PhylPro
1.	HQ439023	KR231951	Unknown (NC_003688)	6.91E-62	9.50E-58	1.79E-64	1.36E-30	1.57E-32	8.43E-37	-
2.	OM948835	OM948855	Unknown (OM948846)	2.06E-34	1.12E-26	7.94E-32	6.81E-24	1.83E-23	4.40E-28	-
3.	OM948841	Unknown (OM948849)	OM948840	6.38E-33	9.35E-28	1.54E-18	2.60E-24	1.32E-18	5.31E-30	-
4.	JQ700306	Unknown (OM948855)	JQ700305	1.92E-26	3.01E-50	3.47E-18	2.57E-28	3.53E-20	4.20E-46	-
5.	OM948839	OM948847	OM948845	7.41E-25	1.62E-20	-	4.76E-19	3.14E-18	8.48E-19	-
6.	OM948845	Unknown (OM948854)	OM948855	2.49E-23	7.48E-19	1.13E-09	1.89E-10	5.63E-09	2.41E-10	-
7.	LC082306	KR231948	EU636992	1.64E-20	7.78E-21	2.09E-21	2.86E-19	6.37E-10	1.26E-26	-

8.	OM948838	OM948847	Unknown (OM948834)	1.12E-19	4.92E-18	3.24E-18	5.58E-18	5.36E-18	2.25E-18	-
9.	OM948840	OM948849	OM948854	6.86E-17	2.40E-18	2.45E-12	2.60E-09	4.35E-09	6.06E-11	-
10.	KR231952	EU636992	KR231944	6.33E-11	8.28E-09	7.62E-09	6.27E-18	7.62E-15	2.23E-11	-
11.	MN688220	Unknown (JQ700305)	JF939813	5.23E-22	9.81E-41	1.93E-14	1.43E-21	2.60E-15	8.08E-34	-
12.	JF939813	OM948856	KR231945	6.99E-18	4.79E-30	-	3.85E-17	3.03E-13	1.65E-25	-
13.	OM948834	OM948851	Unknown (OM948836)	8.11E-18	3.67E-16	1.20E-16	1.92E-12	3.11E-11	3.38E-14	-
14.	JQ700305	LC472499	OM948834	1.95E-31	1.11E-21	1.23E-30	2.03E-18	3.90E-13	1.17E-26	-
15.	KR231949	MG257902	EU636992	5.26E-14	2.08E-15	5.79E-20	2.02E-16	6.93E-16	1.13E-21	-
16.	OM948851	OM948849	OM948836	1.74E-15	4.59E-16	3.83E-14	1.21E-10	2.99E-09	5.92E-09	-
17.	OM948841	Unknown (KR231960)	OM948836	9.47E-14	4.21E-12	3.23E-05	3.64E-15	2.87E-12	7.43E-10	-
18.	OM948841	OM948854	Unknown (OM948855)	8.34E-15	1.79E-12	3.20E-09	8.36E-10	5.18E-10	9.27E-12	-
19.	EU636992	NC_003688	KR231957	1.08E-08	4.47E-06	-	9.74E-14	1.08E-09	4.69E-29	-

20.	OM948846	OM948855	Unknown (NC_003688)	2.19E-11	3.65E-10	4.52E-10	4.36E-13	4.99E-10	2.92E-18	-
21.	GQ221223	KR231951	Unknown (KR231942)	6.59E-13	6.51E-10	5.70E-08	9.39E-11	2.88E-11	9.86E-16	-
22.	JF939814	OM948851	OM948855	5.12E-08	6.14E-09	9.36E-11	4.88E-12	8.86E-10	3.37E-17	-
23.	OM948835	OM948854	OM948849	3.46E-08	9.56E-11	-	1.30E-09	2.94E-10	8.51E-08	-
24.	KR231949	KR231948	MK055337	2.51E-06	7.91E-08	9.66E-09	4.07E-09	1.95E-09	8.51E-21	-
25.	KR231952	KR231956	LC082306	8.03E-09	8.58E-08	4.69E-09	2.00E-06	5.40E-06	5.20E-06	-
26.	MT027103	OM948836	NC_003688	9.13E-07	2.50E-05	7.29E-07	5.96E-07	4.33E-08	2.23E-12	-
27.	JQ700306	MK055337	NC_003688	1.69E-06	5.11E-05	-	7.92E-07	6.68E-06	1.48E-19	-
